# Pre-Bout Standing Body Sway Differs between Adult Boxers Who Do and Do Not Report Post-Bout Motion Sickness

**DOI:** 10.1371/journal.pone.0046136

**Published:** 2012-10-03

**Authors:** Yi-Chou Chen, Ting-Hsuan Hung, Tzu-Chiang Tseng, City C. Hsieh, Fu-Chen Chen, Thomas A. Stoffregen

**Affiliations:** 1 School of Kinesiology, University of Minnesota, Minneapolis, Minnesota, United States of America; 2 Office of Institutional Research, Edgewood College, Madison, Wisconsin, United States of America; 3 Graduate Institute of Sport Coaching Science, Chinese Cultural University, Taipei, Taiwan; 4 College of Well Being, YuanPei University, HsinChu, Taiwan; 5 Department of Recreation Sport and Health Promotion, National Pingtung University of Science and Technology, Pingtung, Taiwan; Nathan Kline Institute and New York University School of Medicine, United States of America

## Abstract

**Background:**

Motion sickness is characterized by subjective symptoms that include dizziness and nausea. Studies have shown that subjective symptoms of motion sickness are preceded by differences in standing body sway between those who experience the symptoms and those who are not. Boxers often report dizziness and nausea immediately after bouts. We predicted that pre-bout standing body sway would differ between boxers who experienced post-bout motion sickness and those who did not.

**Methodology/Principal Findings:**

We collected data on standing body sway before bouts. During measurement of body sway participants performed two visual tasks. In addition, we varied stance width (the distance between the heels). Postural testing was conducted separately before and after participants' regular warm-up routines. After bouts, we collected self-reports of motion sickness incidence and symptoms. Results revealed that standing body sway was greater after warm-up than before warm-up, and that wider stance width was associated with reduced sway. Eight of 15 amateur boxers reported motion sickness after a bout. Two statistically significant interactions revealed that standing body sway before bouts differed between participants who reported post-bout motion sickness and those who did not.

**Conclusions/Significance:**

The results suggest that susceptibility to motion sickness in boxers may be manifested in characteristic patterns of body sway. It may be possible to use pre-bout data on postural sway to predict susceptibility to post-bout motion sickness.

## Introduction

Boxing is characterized by high intensity cardiovascular activity, by intense concentration and, in many cases, by blows to the head. After bouts, boxers often experience headache, confusion, memory difficulties, fatigue, attention and concentration difficulties, and sleep disturbances that can persist for hours, days, weeks, or months [1]. Immediately after bouts, boxers often experience dizziness and nausea [2]. These latter symptoms classically are associated with motion sickness and, indeed, boxers often refer to their acute post-bout symptoms as *motion sickness*. In the present study, our focus was on relations between motion sickness and standing body sway in adult boxers.

### Postural sway and motion sickness

People often experience motion sickness when exposed to simulation and virtual environment systems. Examples include video games [3]–[5], video projection systems [6]–[7], head-mounted displays [8]–[9] and flight simulators [10]–[12]. Exposure to simulators and virtual environments is associated with generalized increases in postural sway [6], [10]–[11], [13]. That is, postural sway measured after using one of these systems differs from sway measured before exposure to the system. Typically, it is assumed that both motion sickness and postural sway effects are caused by the fact of being exposed to a virtual environment. However, several studies have revealed differences in postural activity between participants who (later) reported motion sickness and those who did not. Differences have been observed in the spatial magnitude of postural sway [12], [14], with greater movement magnitude among participants who later reported motion sickness. Differences have also been observed in the temporal dynamics of postural sway, with greater temporal structure or self-similarity among participants who later reported motion sickness [15]. These effects are not limited to virtual environments. Nachum et al. [16] measured participants' standing sway before and after a sea voyage and related these data to the incidence of mal de debarquement (motion sickness that occurs after returning to land from a ship). Prior to a sea voyage, postural activity differed between sailors who reported mal de debarquement after sailing and those who did not. These effects provide the empirical motivation for the present study.

Motion sickness-like symptoms, such as nausea and dizziness, characterize many conditions that typically are not considered to be related to motion sickness, such as altitude sickness [17], vertigo [18]–[19], and morning sickness in pregnancy [20], as well as boxing. Stoffregen [21] argued that there might be differences in postural sway between persons who are susceptible to these maladies and persons who are not susceptible. The documented relation between postural sway and the subsequent experience of visually induced motion sickness suggests that data on body sway might be used to predict susceptibility to motion sickness in individuals [21]. In the present study our primary aim was to test the hypothesis that postural sway before a bout would differ between boxers who experienced post-bout motion sickness and those who did not.

### Modulating factors

Under controlled manipulations of stance width (the distance between the heels) body sway tends to be greater when the feet are close together, and less when the feet are farther apart [22]–[23]. Variations in stance width can also alter the temporal dynamics of sway [24], [25]. When asked to stand comfortably, healthy adults typically place their heels about 17 cm apart [26]. However, self-selected stance width can change according to circumstances. During pregnancy women tend to select wider stance, that is, they increase the distance between the feet [27]. We also vary stance width rapidly in different situations. For example, mariners adopt wider stance width at sea than they do on land [28], and baseball players typically adopt a wide stance when batting. Of greater relevance to the present study, boxers typically adopt a wide stance in the ring. This habitual, task-specific choice may influence relations between stance width and standing body sway. An important additional factor is the experimental finding that wider stance reduces susceptibility to visually induced motion sickness [15]. In light of these factors we elected to manipulate stance width, and we predicted that wider stance would lead to reduced sway in boxers.

In the general population, standing body sway is influenced by variations in visual and cognitive tasks such as auditory reaction time, or visual task difficulty [29]. For example, sway magnitude is often reduced during performance of demanding tasks, such as reading, relative to sway during less demanding tasks, such as looking at a blank target [24], [30]–[32]. Studies of standing body sway in athletes have evaluated eyes open and eyes closed conditions but have not included variations in visual tasks [33]–[36]. We hypothesized that the magnitude and self-similarity of postural sway would be reduced during performance of a demanding visual task in boxers when tested before a bout.

### The present study

In the present study, our primary objective was to determine whether standing body sway measured before a bout would differ between boxers who reported post-bout motion sickness and those who did not. We measured standing body sway before boxers entered the ring. After boxers completed their bout, we evaluated subjective symptoms that typically are associated with motion sickness. We predicted that patterns of pre-bout postural sway would differ between boxers who later experienced motion sickness and those that did not.

We measured body sway in the absence of any external source of motion (i.e., there was no mechanical perturbation, such as occurs in moving platform posturography [33]–[34]). In addition, unlike many previous studies we did not ask participants to stand “as still as possible” [36]–[37]; rather, we instructed participants to stand comfortably.

## Methods

### Ethics statement

The research protocol was approved in advance by the YuanPei University IRB. Prior to data collection, we obtained informed consent from each participant.

Testing was conducted at the Contender Fitness Boxing Club, New Taipei City, Taiwan during a national boxing competition for club level boxers. In Taiwan, *club level* refers to amateur boxers whose age and training background are compatible with rules of the World Series of Boxing of the International Boxing Association (known as AIBA).

### Participants

Seventeen boxers participated. Due to time pressure relating to the schedule of bouts, two participants were not able to participate in postural testing and, for this reason, were deleted from our analyses. Accordingly, our sample included 15 individuals. All were male. They varied in age from 18–34 years (mean = 25.6 years, SD = 5.1 years), in height from 160–186 cm (mean = 173.5 cm, SD = 7.9 cm), and in weight from 53–106 kg (mean = 72.8 kg, SD = 15.7 kg).

### Apparatus

Data on postural activity were collected using a force plate (AccuswayPlus, AMTI). We collected data on the kinematics of the center of pressure, sampled at 60 Hz in the AP and ML axes.

### Procedure

We evaluated motion sickness incidence and symptoms using the Simulator Sickness Questionnaire, or *SSQ* [38]. We used a modified version of the SSQ. The modification consisted of the addition of one question: *Are you motion sick?* In responding to this question, participants were required to circle either *yes* or *no*. For this study the SSQ was translated into Chinese.

Data were collected in relation to each individual's first bout in the national competition. All data were collected on the day of the bout. Prior to their bout, boxers went through a warm-up routine, usually consisting of 5 minutes of light jogging, extensive stretching, and “mitten drills” in which they practiced different types of punches. The total duration of the warm-up was approximately 30 minutes. The warm-up increased heart rate and respiration and for this reason might influence postural sway which, in turn, might influence relations between pre-bout sway and post-bout subjective symptoms. To account for this possibility we measured postural sway before warm-up and again after warm-up.

Before each participant went through his regular warm-up routine he completed the informed consent procedure, the first SSQ and the first session of postural testing. After completing the warm-up each participant completed the second SSQ and the second session of postural testing.

Postural testing consisted of 6 trials, each 60 s in duration, standing on the force plate. We used a 2 (Inspection vs. Search)×3 (Stance Width = 5 cm vs. 17 cm vs. 30 cm) design with one trial per session (before vs. after warm-up) in each of six conditions for a total of 12 trials per participant. Within each session the order of conditions was counterbalanced across participants.

Visual targets used during postural testing were identical to those used by Stoffregen et al. [31], and consisted of sheets of white paper 13.5 cm×17 cm mounted on rigid cardboard. In the Search task the target was one of four blocks of English text, each consisting of 13 or 14 lines of text printed in a 12-point sans serif font. Before each trial the participant was given a target letter (A, R, N, or S) and asked to count the number of times the target letter appeared in the block of text. At the end of each trial, the participant reported the number of letters counted. The Search task resembled the King-Devick test, which has been used to assess cognitive consequences of head trauma in boxers [39]. In the Inspection task, the target consisted of a blank sheet of white paper; participants were instructed to keep their gaze within the borders of the target. The Inspection task was similar to “quiet stance” conditions used in previous studies [29] and can be considered a control condition for the Search task.

Bouts consisted of three rounds (3 minutes per round), and could be terminated early in the event of knockout (KO) or technical knockout (TKO). The third SSQ was administered during the “cool down” period, 10 to 20 minutes after the bout.

### Analysis of Postural Data

We separately evaluated the magnitude and temporal dynamics of postural activity. Magnitude measures, such as positional variability, velocity, and range, provide information about the size or spatial extent of movement (e.g., “by how many centimeters do COP data points tend to differ from each other?”). Magnitude measures, by their nature, tend to eliminate or discard the temporal structure of movement data, that is, how the measured quantity varies in time (e.g., “to what extent does COP displacement at time A resemble displacement at time B?”). Analyses that preserve information about the temporal structure of data on human movement (that is, analyses of the temporal dynamics of movement) are increasingly common [40]–[41], and can reveal changes in the temporal structure of postural activity in response to variations in visual tasks [42]. We assessed movement dynamics using detrended fluctuation analysis, or *DFA*. DFA describes the relation between the magnitude of fluctuations in postural motion and the time scale over which those fluctuations are measured [43]. DFA has been used in several studies of the control of stance [35], and in our own research on visually induced motion sickness [4], [7], [15]. We did not integrate the time series before performing DFA. We conducted inferential tests on α, the scaling exponent of DFA, as derived from the COP data. The scaling exponent is an index of long-range autocorrelation in the data, that is, the extent to which the data are self-similar over different time-scales. Postural sway in healthy adults tends to be non-stationary, typically yielding 1.0>α>1.5 [41].

We conducted 2×2×2×3 repeated measures ANOVAs on factors Group (Sick vs. Well), Task (Inspection vs. Search), Warm-up (Before warm-up vs. After warm-up), and Stance Width (5 cm vs. 17 cm vs. 30 cm). The dependent variables were the positional variability of the center of pressure, and α of DFA. Separate analyses were conducted for postural activity in the AP and ML axes. To accommodate any violations of the ANOVA sphericity assumption, we used the Greenhouse-Giesser correction [44], which adjusts the number of degrees of freedom used for individual comparisons in the ANOVA in response to violations of sphericity. Where appropriate we report the fractional degrees of freedom that characterize this correction. For statistically significant effects we used the partial η^2^ statistic as a measure of effect size.

## Results

Participants (and their coaches) indicated that they regularly trained and sparred at their local boxing clubs and participated in local, club level competitions. None of the participants had competed in any boxing tournament in the previous month. In the two weeks preceding the national competition all competitors had reduced schedules of sparring. This less intensive level of practice was maintained until the day of the competition. Thus, none of the participants had experienced a concussion or loss of consciousness during the two weeks prior to their participation in our study.

None of the participants were diagnosed with a concussion following their participation in the study.

### Visual performance

**Figure 1:**
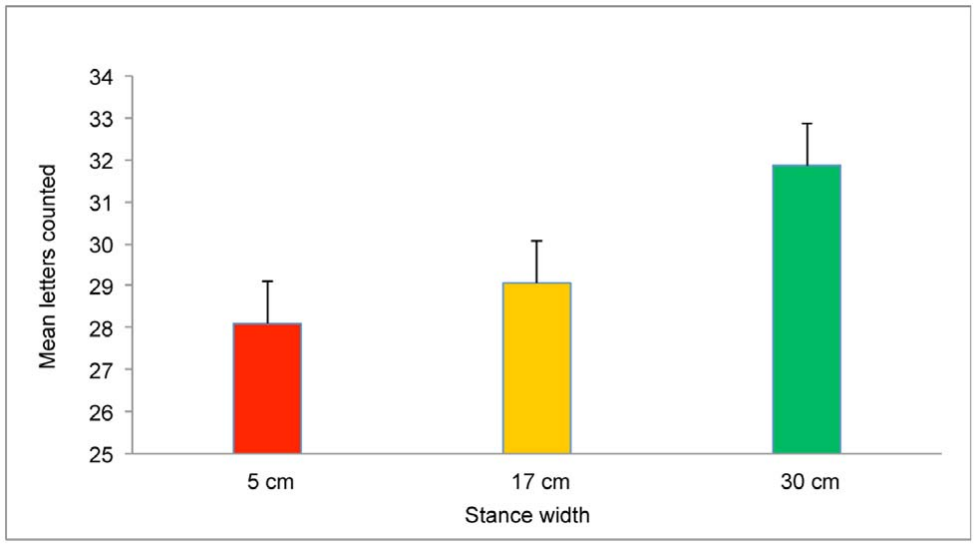
Performance on the Search task (mean letters counted per trial) as a function of stance width. The error bars represent standard error of the mean.

There was no measure of performance for the Inspection task. Following previous studies, we took for granted that participants maintained their gaze within the boundaries of the blank target [31]. For the Search task, the dependent variable was the number of target letters that S reported at the end of each trial. We conducted a 2×2×3 repeated measures ANOVA on factors Group (Sick vs. Well), Time (Before warm-up vs. After warm-up), and Stance Width (5 cm vs. 17 cm vs. 30 cm). Visual performance was influenced by stance width, F(2,26) = 3.64, *p* = .04. As shown in Figure 1, the number of letters counted was positively associated with greater stance width. There were no other significant effects.

### Subjective symptoms

Before and after warm-up, each participant stated that they were not motion sick. After their bout, 8 participants stated that they were not motion sick. Seven of 15 (47%) stated that they were motion sick (should sum. Data on wins and losses are presented in Table 1.

**Table 1:** Bout outcomes for boxers in the Well and Sick groups.

	Total	Unanimous	Split	TKO-1	TKO-2	TKO-3
Well wins	6	3	1	1	0	1
Well losses	2	2	0	0	0	0
Sick wins	3	0	1	0	1	1
Sick losses	4	2	0	0	1	1

Bouts were evaluated by three judges. Unanimous: All three judges concurred on the winner. Split: Two judges agreed on the winner. Some bouts ended with a technical knockout, or TKO. In these bouts no judges' decision was needed. TKO-1,2,3 indicates the TKOs that occurred in the first, second, or third round.

**Figure 2:**
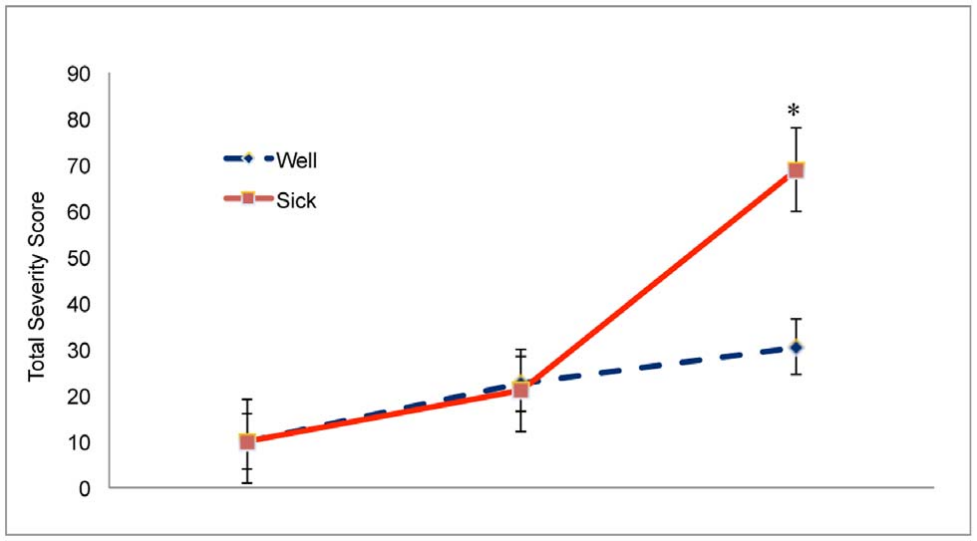
Severity of motion sickness symptoms as measured using the Total Severity Score of the Simulator Sickness Questionnaire. *, post-hoc difference between Well and Sick groups after completion of bouts, *p*<.05. The error bars represent standard error of the mean.

Data on symptom severity are summarized in Figure 2. In evaluating the severity of symptoms we used the Total Severity Score, which we computed in the recommended manner [38]. The third SSQ was completed approximately 15 minutes after each participant's bout. Before warm-up, SSQ scores did not differ between participants who later reported motion sickness and participants who did not, *U* = 31, *p* = .340. After warm-up, SSQ scores did not differ between participants who later reported motion sickness and participants who did not, *U* = 30, *p* = .386. After their bouts, SSQ scores were higher among participants in the Sick group than in the Well group, *U* = 42, *p*<.05.

### Postural activity

**Figure 3:**
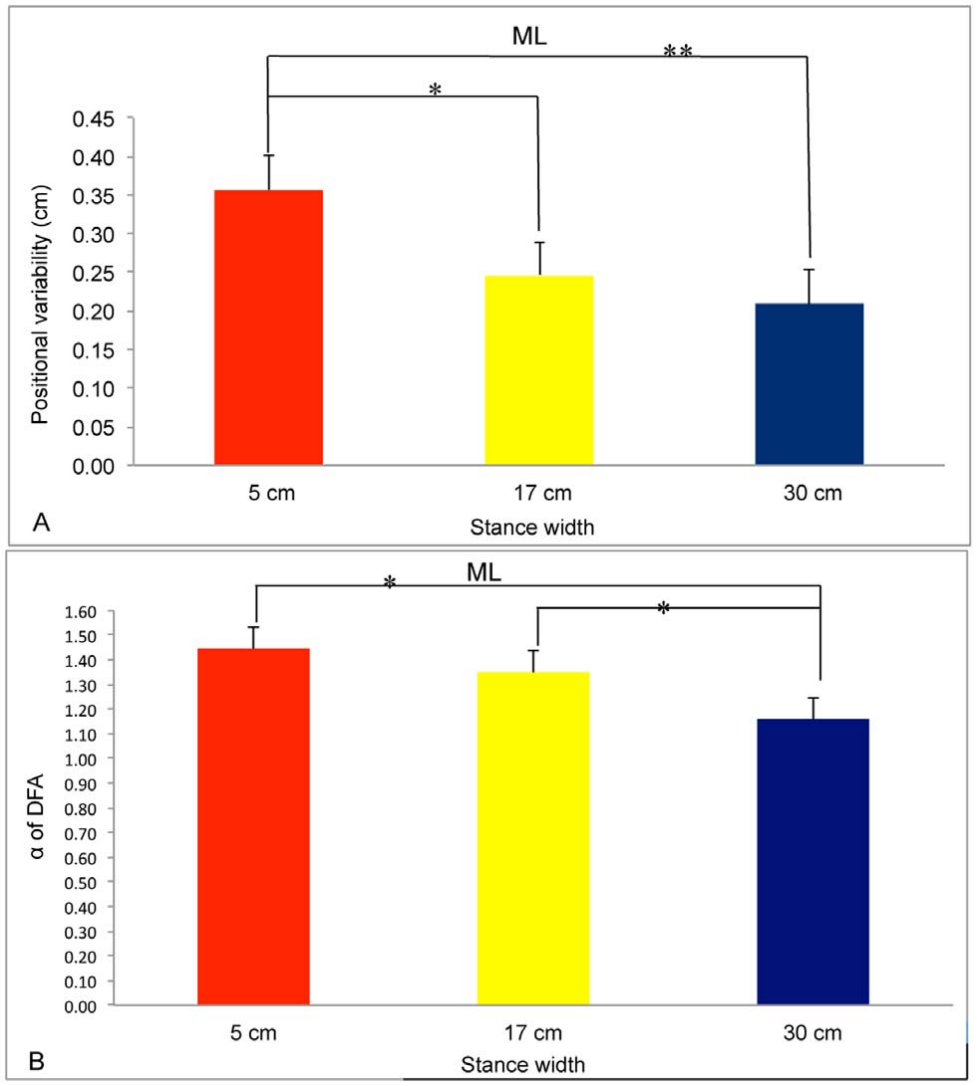
Postural activity in the ML axis as a function of stance width. A. Positional variability of the COP. B. Self-similarity of COP positions as quantified by α, the scaling exponent of detrended fluctuation analysis. *, *p*<.05; ** *p*<.01. The error bars represent standard error of the mean.

**Figure 4:**
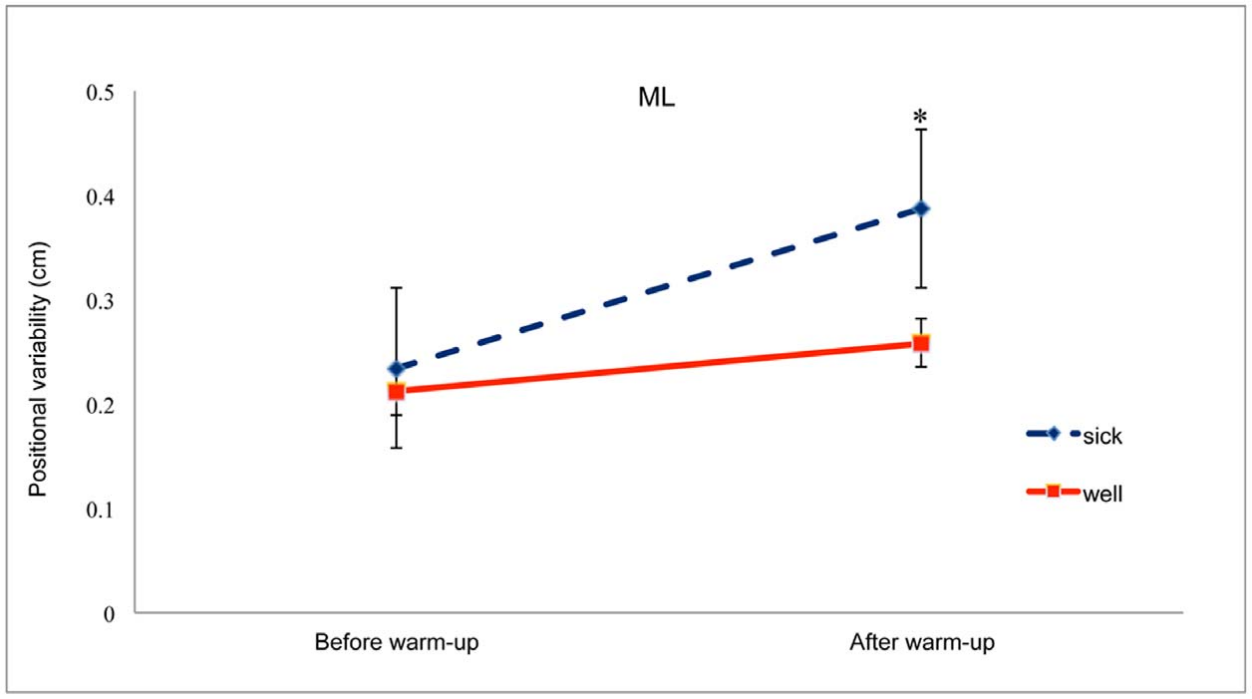
Positional variability of the COP in the ML axis before and after warm-up for the Well and Sick groups. *, post-hoc difference between Well and Sick groups after boxers completed their warm-up routine, *p*<.05. The error bars represent standard error of the mean.

**Figure 5:**
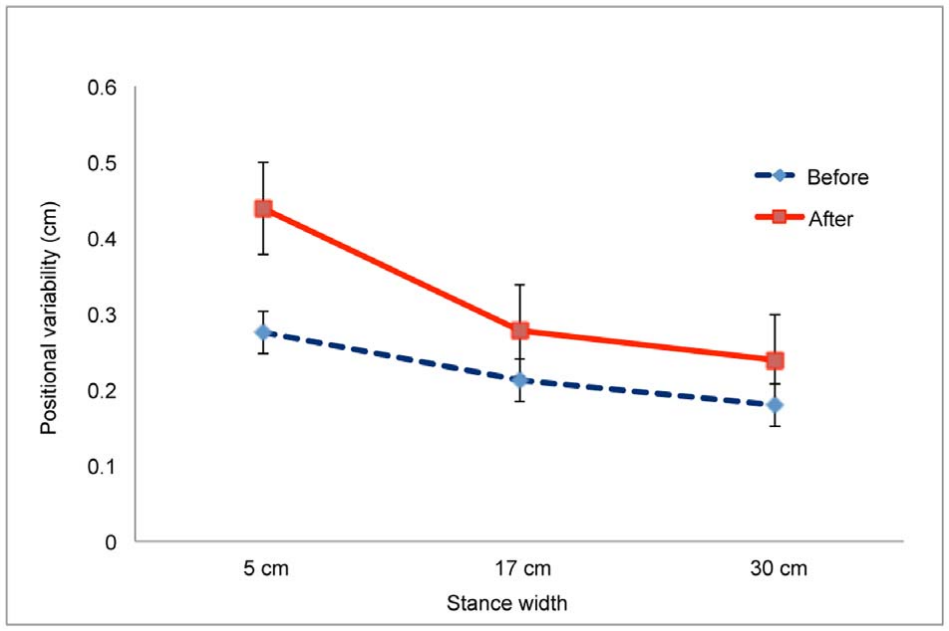
Positional variability of the COP in the ML axis before and after warm-up as a function of stance width. The error bars represent standard error of the mean.

For positional variability in the ML axis we found a significant main effect of Warm-up, F(1,13) = 51.87, *p*<.001, partial η^2^ = 0.800. Positional variability after warm-up (mean = 0.323, SD = 0.019) was greater than before warm-up (mean = 0.223, SD = 0.023). The main effect of Stance Width was also significant, F(1.22,26) = 22.98, *p*<.001, partial η^2^ = 0.639 (Figure 3a). Post-hoc tests revealed that each stance width differed from each of the other two stance widths, each *p*<.008. There was a significant Group×Warm-up interaction, F(1,6) = 14.94, *p* = .002, partial η^2^ = 0.535 (Figure 4). Post-hoc comparisons revealed that, after warm-up, positional variability was greater for the Sick group than for the Well group, *p*<.001. Finally, the Warm-up×Stance Width interaction was significant, F(1.55,20.13) = 7.42, *p* = .006, partial η^2^ = 0.363 (Figure 5). Post-hoc comparisons revealed that stance width had a significant influence on sway after warm-up but not before warm-up, *p*<.05, confirming our prediction.

**Figure 6:**
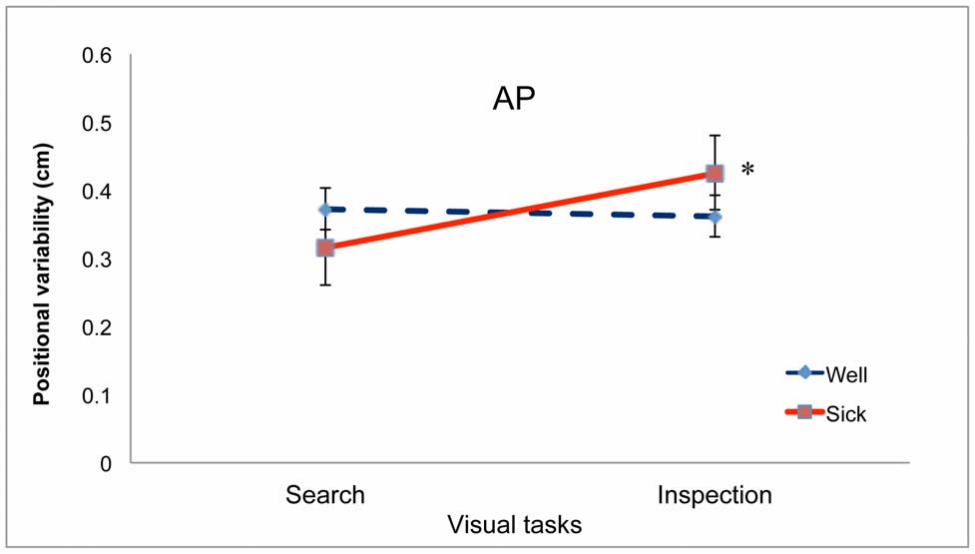
Positional variability of the COP in the AP axis during performance of the Inspection and Search tasks for the Well and Sick groups. *, post-hoc effect for the Sick group, sway was reduced during performance of the Search task, relative to sway during performance of the Inspection task, *p*<.05. The error bars represent standard error of the mean.

**Figure 7:**
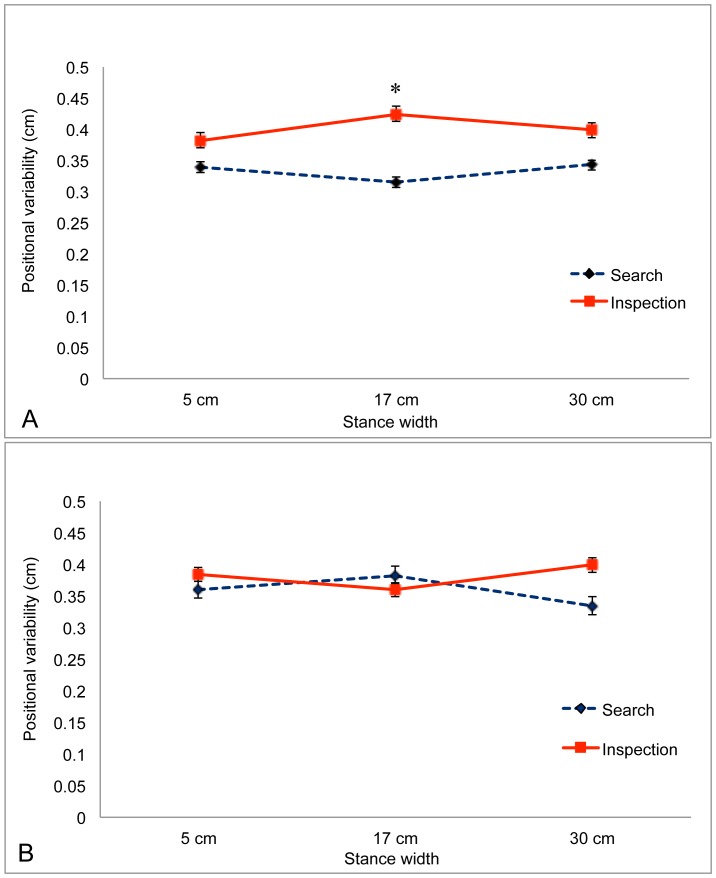
Positional variability of the COP in the AP axis as a function of stance width during performance of the Inspection and Search tasks. A. Before warm-up. B. After warm-up. *, post-hoc effect showing that before warm-up sway was reduced during performance of the Search task, relative to sway during performance of the Inspection task, when stance width was 17 cm, *p*<.05. The error bars represent standard error of the mean.

For positional variability in the AP axis we found no significant main effects. There was a significant interaction between Group and Task, F(1,13) = 6.69, *p* = .023, partial η^2^ = 0.340, which is illustrated in Figure 6. Post-hoc tests revealed that for boxers in the Sick group sway was reduced during the Search task, relative to sway during the Inspection task, *p*<.05, whereas for boxers in the Well group sway did not differ as a function of visual task. In addition, there was a significant 3-way interaction between Task, Warm-up, and Stance Width, F(1.75,22.69) = 4.69, *p* = .023, partial η^2^ = 0.265, which is illustrated in Figure 7. Post-hoc tests revealed that before the warm-up (Figure 7A) positional variability was reduced during performance of the Search task (relative to sway during performance of the Inspection task) when stance width was 17 cm, *p* = .018.

For α of DFA in the ML axis we found a significant main effect of stance width, F(1,1.351) = 77.13, *p*<.001, partial η^2^ = .847 (Figure 3b). Post-hoc tests revealed that each condition differed from both of the others. The effect of stance width on α in the ML axis resembled that reported by Stoffregen et al. ([15], their figure 7) for healthy undergraduates (mean age = 20 years). For α of DFA in the AP axis there were no significant effects.

## Discussion

Using a simple, non-invasive testing protocol, we measured the standing body sway of amateur boxers before they entered the ring for a competitive bout. After completing the bout boxers reported the presence and severity of motion sickness. Measures of standing body sway taken before the bout differed significantly between boxers who did and did not report motion sickness after the bout as a function of time (before warm-up versus after warm-up) and as a function of visual task (Inspection vs. Search). The present study appears to be the first to document relationships between pre-bout postural sway and post-bout motion sickness.

### Boxers relative to the general population

Postural sway often is reduced during performance of difficult visual tasks, relative to sway during performance of easy visual tasks [29], [31]. In previous studies body sway has been measured in the absence of any physical exertion and with participants adopting their preferred stance width. In the present study we found the same effect under comparable conditions (i.e., before warm-up, with stance width = 17 cm; Figure 7A). In the general population, the positional variability of postural sway is inversely related to stance width [22]–[23]. In the present study we found the same effect in the body's ML axis (Figure 3). In these two ways the body sway of boxers in the present study resembled effects documented in the general population. While stance width influenced postural sway, it also influenced performance on the visual search task: Wider stance was associated with increases in the reported count of target letters (Figure 1). Yu et al. [24] observed a similar effect in the context of visual performance among mariners on a ship at sea.

### Postural effects of pre-bout warm-up routine

Body sway differed before and after the pre-bout warm-up routine. As expected, the positional variability of the COP was greater after warm-up than before warm-up, but this was true only for sway in the ML axis. Warm-up also increased the influence of stance width on the positional variability of the COP in the ML axis (Figure 5). These effects can be explained by the increased physiological arousal that occurs during warm-up. By contrast, in the AP axis positional variability did not exhibit an overall increase following warm-up; rather, the effects of warm-up were modulated by stance width and visual task (Figure 7). Warm-up influenced the positional variability of sway but had no effect on the temporal dynamics of sway; that is, warm-up increased the magnitude of sway not its temporal structure.

### Pre-bout postural sway and post-bout motion sickness

Our principal hypothesis was that postural sway before a bout would differ between boxers who reported motion sickness after the bout those who did not. This prediction was confirmed for the positional variability of the COP. In previous research on the general population of healthy adults we have examined relations between visual motion, postural sway, and motion sickness [12], [15]. In those studies, we found that changes in postural sway occurred among individuals who reported visual motion sickness, and that these changes began before the onset of subjective symptoms of visual motion sickness. In the present study we found similar effects: Postural sway differed between boxers who did and did not report motion sickness, and these changes existed before boxers entered the ring.

After warm-up, the positional variability of the COP in the ML axis was greater for boxers who reported post-bout motion sickness than for boxers who did not (Figure 4). One possible interpretation of this effect is that participants in the Sick group were less able than their Well peers to compensate for postural effects of physiological arousal. An alternative interpretation is that, after warm-up, participants in the Sick group relaxed their criterion for “comfortable” stance.

We also found that our manipulation of visual tasks influenced the positional variability of the COP in the AP axis for Sick boxers, but not for Well boxers (Figure 6). Participants in the Sick group tended to reduce their sway during performance of the Search task, relative to sway during performance of the Inspection task. Modulation of postural sway in response to different visual tasks has been reported in many studies [29], [45]. Separately, the control of standing body sway can be affected by clinical conditions such as aging [32] and Parkinson's disease [46]. Clinical conditions can also influence the task specific modulation of standing body sway. For example, children with autism spectrum disorder modulate their sway in response to variations in visual tasks [30], but children at risk for developmental coordination disorder do not [47]. The present study provides the first evidence that task-specific variation in postural sway may be related to individual differences in susceptibility to motion sickness.

### Causal factors

Dizziness, nausea and vomiting are common acute symptoms of concussion [2], [48]. In addition to these subjective symptoms concussion also is associated with changes in standing body sway, both in the immediate aftermath of head trauma [2] and up to several months later [33]–[35], [49]. Boxing is widely associated with concussion [39], [50]. Given the results of the present study these facts suggest that pre-bout postural sway may be related to an individual's susceptibility to concussion. In future research it will be important to examine possible relations between pre-bout postural sway, post-bout motion sickness, and concussion. It may be possible to use pre-bout data on postural sway as a predictor of susceptibility to boxing-related concussion.

We were not able to record the number or severity of blows to the head during bouts. It is possible that participants in the Sick group sustained more blows to the head, or more severe blows to the head, than participants in the Well group. Such a relation would be expected if post-bout motion sickness were causally related to head trauma (either concussive or sub-concussive) experienced during individual bouts. We predict that pre-bout postural sway would differ between boxers who did and did not experience post-bout motion sickness when controlling for the number and severity of blows to the head experienced by each boxer. Similarly, a person's experience of post-bout motion sickness might vary from bout to bout, that is, a person who did not experience motion sickness after one bout might experience it after a subsequent bout, and vice versa (as one example, if participants who did not experience motion sickness in the present study were paired to fight each other, then they might experience motion sickness in this second bout). In particular, known long-term effects of concussion on body sway [33]–[35], [49] indicate lingering effects of concussion on motor control which, in turn, might increase susceptibility to motion sickness-like symptoms in subsequent bouts. It would be interesting to conduct a longitudinal study evaluating pre-bout postural sway and post-bout motion sickness in the same individuals over a succession of bouts. Such a study would make it possible to determine whether relations between pre-bout postural sway and post-bout motion sickness persist across bouts.

It is important to recall, however, that in the present study none of the participants were diagnosed with a concussion following their participation. Motion sickness is a common consequence of athletic concussion [2], [48]; however, athletes sometimes experience motion sickness in the absence of concussion, and in the absence of any head trauma [51], [52]. Thus, in the present study it is possible that post-bout motion sickness occurred in the absence of significant head trauma. The preceding possibilities are not mutually exclusive; that is, it may be that post-bout motion sickness has multiple causal factors, including but not limited to head trauma sustained during the bout. This is an area for future research.

## Conclusion

We conducted the first assessment of the quantitative kinematics of standing body sway in boxers. We used data on pre-bout postural sway in a prospective manner. We showed that before entering the ring there were differences in standing body sway between boxers who experienced post-bout motion sickness and those who did not. These effects suggest the possibility that objective, non-invasive measures of postural control might be helpful in evaluating susceptibility to boxing-related concussion.
